# A Regulated Temperature-Insensitive High-Voltage Charge Pump in Standard CMOS Process for Micromachined Gyroscopes

**DOI:** 10.3390/s19194149

**Published:** 2019-09-25

**Authors:** Xiang Li, Rui Li, Chunge Ju, Bo Hou, Qi Wei, Bin Zhou, Zhiyong Chen, Rong Zhang

**Affiliations:** Department of Precision Instruments, Tsinghua University, Beijing 100084, China; li-x07@mails.tsinghua.edu.cn (X.L.); lr17@mails.tsinghua.edu.cn (R.L.); jcg17@mails.tsinghua.edu.cn (C.J.); houb15@mails.tsinghua.edu.cn (B.H.); rongzh@mail.tsinghua.edu.cn (R.Z.)

**Keywords:** high-voltage generation, regulated charge pump, temperature-insensitive, triple-well NMOS transistor, standard CMOS process

## Abstract

Micromachined gyroscopes require high voltage (HV) for actuation and detection to improve its precision, but the deviation of the HV caused by temperature fluctuations will degrade the sensor’s performance. In this paper, a high-voltage temperature-insensitive charge pump is proposed. Without adopting BCD (bipolar-CMOS-DMOS) technology, the output voltage can be boosted over the breakdown voltage of n-well/substrate diode using triple-well NMOS (n-type metal-oxide-semiconductor) transistors. By controlling the pumping clock’s amplitude continuously, closed-loop regulation is realized to reduce the output voltage’s sensitivity to temperature changes. Besides, the output level is programmable linearly in a large range by changing the reference voltage. The whole circuit has been fabricated in a 0.18-μm standard CMOS (complementary metal-oxide-semiconductor) process with a total area of 2.53 mm${}^{2}$. Measurements indicate that its output voltage has a linear adjustable range from around 13 V to 16.95 V, and temperature tests show that the maximum variations of the output voltage at $-40∼80{\;}^{\circ }C$ are less than 1.1%.

## 1. Introduction

Micromachined gyroscopes are widely used for sensing rotation in many applications, such as integrated navigation, automotive safety and portable devices [1,2]. Angular rate information is sensed by Coriolis effect on the vibrating mass of micro gyroscope with electrical actuation [3,4]. Increasing vibration amplitude of gyroscope is beneficial to improve its resolution, which requires high voltage driving (HV) [5]. HV also can be utilized in gyroscope circuits for quadrature compensation and electrostatic force balance, to acquire better accuracy, broader measurement range and larger scale factor [6,7,8,9]. As there has been a trend of miniaturization and integration of the electronic interface along with the development of micro gyroscopes, HV generator is also expected to be built on-chip to mitigate the complexity of the circuit and reduce power consumption [10,11].

Charge pump is commonly used as a HV generator on chip [12]. The HV actuating the gyroscope needs to be robust in different operating conditions and programmable in a large range to remain a stable vibrating state [13,14]. Temperature insensitivity is a key factor in developing practical micro-gyroscope system into high-precision application area [15]. For an unregulated charge pump, environmental factors such as temperature fluctuations will cause non-negligible variations at the charge pump’s output terminal. Since the HV is imposed on the driving electrodes of the gyroscope, the HV variation will directly affect the vibration amplitude of the sensor and consequently be sampled at the sensing nodes, which lowers the gyroscope’s accuracy. Therefore, closed-loop operation is required to reduce the output voltage fluctuation. The regulation can be achieved by controlling the frequency of clock signal [11,16,17], which induces a voltage-controlled oscillator (VCO). There are also digital approaches reported to realize closed-loop charge pumps [18,19]. However, non-linearity is a challenge to the charge pump’s performance as designing with these clock frequency-related controlling schemes.

Generally, a charge pump’s output voltage is limited by the breakdown voltage of the well/substrate diodes, and to solve this problem, previous works applied silicon-on-insulator (SOI) process [20] and polysilicon diodes on shallow trench isolation (STI) layer [21], which increased fabrication cost and circuit design complexity. Moreover, utilizing HV process is another choice to attain sufficient voltage level [11,22]. However, compared to utilizing HV CMOS process, implementing HV generation in a low-voltage standard CMOS process is more affordable with lower cost. The attainable maximum output voltage in standard CMOS process is limited by several breakdown effects, which may occur at n-well/substrate diode or poly-poly capacitor. To realize sufficient voltage level in standard CMOS process, Zhang and Llaser [23] utilized a hybrid charge pump, composed of two Dickson charge pumps and a stacking charge pump to overcome the capacitor breakdown voltage and achieved the output up to the n-well/substrate breakdown limit. The authors of [19,24,25] overcame the limitation of p-n junction breakdown voltage by using triple-well NMOS devices.

In this paper, a high-voltage temperature-insensitive charge pump circuit for micro-gyroscopes is proposed. To realize output voltage regulation, a closed-loop scheme is established via adjusting the amplitude of pumping clock, which can be regarded as a linear and continuous controlling method. And the output voltage can be modified linearly in a large interval by changing the reference. The charge pump has an output surmounting the breakdown voltage of a reverse-biased p-n junction with the application of triple-well NMOS transistors, and is fabricated in a 0.18-μm standard CMOS process without special HV devices.

## 2. Design of the Charge Pump

### 2.1. Charge Pump Architecture

**Figure 1:**
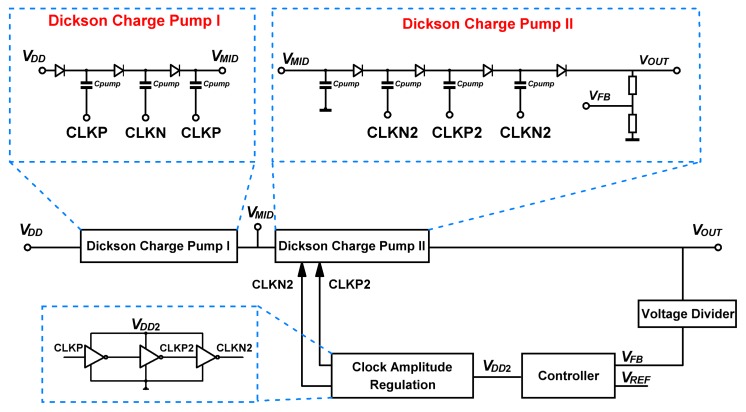
Block diagram of the proposed charge pump.

The block diagram of the proposed charge pump is shown in Figure 1. The core part of the circuit is constituted by two stages of Dickson charge pumps [26]. The first sub-charge pump (Dickson charge pump I) has three stages, driven by differential clocks CLKP and CLKN, which oscillate between ground and DC (direct current) source voltage $V_{DD}$. It generates a voltage $V_{MID}$, which cannot exceed the breakdown voltage of p-n junction. The second sub-charge pump (Dickson charge pump II) is added to boost the output voltage to a higher level, with four stages actuated by regulated clocks CLKP2 and CLKN2. The output voltage ($V_{OUT}$) is then sampled by a resistive voltage divider, getting $V_{FB}=V_{OUT}/M$, and $V_{FB}$ is compared with a reference voltage ($V_{REF}$). The result of comparison is fed into a P-controller to generate the up level of CLKP2 and CLKN2. By this clock amplitude regulation, the output voltage is controlled to a fixed value.

**Figure 2:**
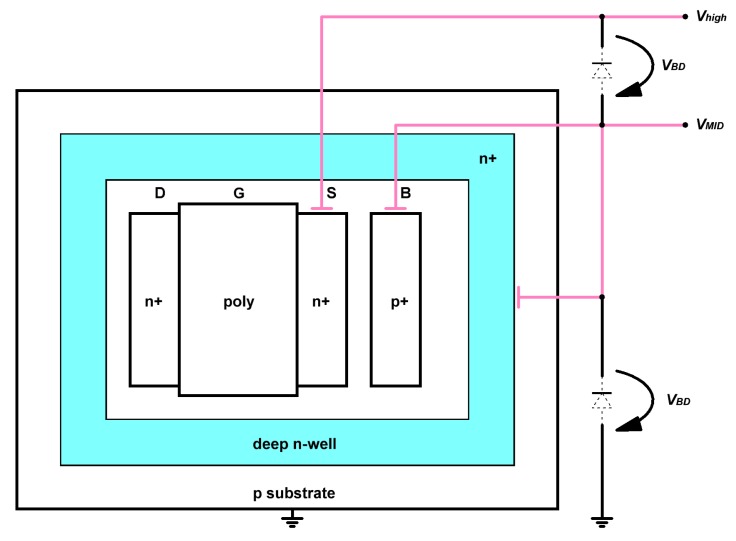
Diode structure built with deep n-well for the Dickson charge pump II [27].

The output voltage of the Dickson charge pump II is higher than the well/substrate breakdown voltage, so the conventional diodes should not be utilized in the second sub-stage to avoid destroying the circuit. Planting the diodes in a deep n-well is an approach to overcome the single p-n junction limit and supports a maximum output level up to twice the breakdown voltage [24,27]. This structure is shown in Figure 2. The deep n-well is lifted up to the output voltage $V_{MID}$ of the first sub-stage, and the body potential of the NMOS in the well is also biased to $V_{MID}$ level. Then, the maximum voltage of the source terminal of this NMOS can be as high as $V_{MID}$ plus a breakdown voltage, which is around 18 V in a standard 0.18-μm CMOS process.

**Figure 3:**
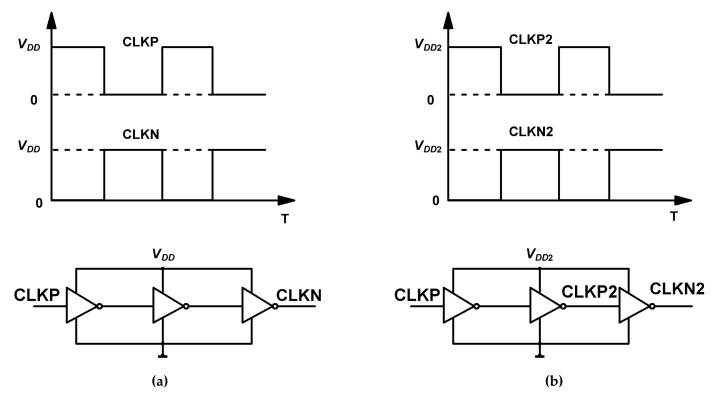
Clocks generation for the two sub-charge pumps: (**a**) clocks for the Dickson charge pump I; and (**b**) clocks for the Dickson charge pump II.

Figure 3 shows the pumping clocks’ generation in the two sub-charge pumps. For the Dickson charge pump I, the amplitude of the non-overlapping clocks CLKP and CLKN is determined by power-supply ($V_{DD}$) and input off-chip. However, for the Dickson charge pump II, the amplitude of the non-overlapping clocks CLKP2 and CLKN2 is determined by the P-controller’s output voltage $V_{DD2}$, which is discussed in detail in the next subsection.

### 2.2. Closed-Loop Regulation

**Figure 4:**
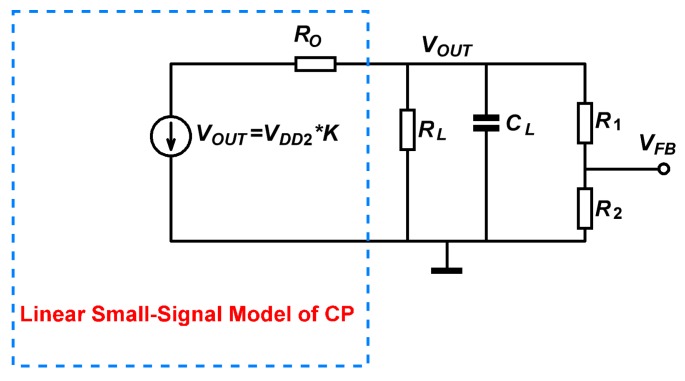
Linear small-signal model of the proposed charge pump.

Closed-loop regulation is necessary to generate stable HV source for micro-gyroscopes. The charge pump can be modeled as a linear voltage source across the pump’s output resistance ($R_{o}$) [28], as shown in Figure 4. The value of the voltage source ($V_{CP}$) is determined by $V_{DD2}$, and it can be expressed as follows

(1) $$V_{CP}=N_{1}\left(V_{DD}-V_{t}\right)+N_{2}\left(V_{DD2}-V_{t}\right)$$

where $N_{1}$ and $N_{2}$ are the number of pumping stages in sub-Dickson charge pump I and II, respectively. Under small-signal assumption, $V_{CP}$ can be modeled as

(2) $$V_{CP}=V_{DD2}\cdot K$$

and *K* is the linear factor [21],

(3) $$K\propto N_{2}\frac{C}{C_{fringe}+C}$$

where $C_{fringe}$ represents total fringe capacitance present at the node (from $C_{GS}$, $C_{SB}$, etc.). Besides, $R_{L}$ and $C_{L}$ are the load of the charge pump.

Then, using the model in Figure 4, the transfer function of the charge pump can be derived as

(4) $$H_{CP}\left(s\right)=\frac{V_{OUT}\left(s\right)}{V_{CP}\left(s\right)}=K_{CP}\frac{1}{\left(R_{o}\|R\right)C_{L}s+1}$$

where

(5) $$R=\left(R_{1}+R_{2}\right)\|R_{L}$$

and

(6) $$K_{CP}=\frac{R}{R_{o}+R}$$

Besides, resistors $R_{1}$ and $R_{2}$ compose the voltage divider, and $V_{FB}$ is the divided voltage result. Because the load capacity of the charge pump is weak, the resistors should be large, and in this work, $R_{1}$ and $R_{2}$ are 119.0304 MΩ and 12.5005 MΩ, respectively. Thus, the divider’s coefficient is

(7) $$H_{FB}=\frac{V_{FB}}{V_{OUT}}=\frac{R_{2}}{R_{1}+R_{2}}$$

Since all the resistors are of the same kind, the proportion $H_{FB}$ hardly changes along with temperature. The time constant of the open loop from $V_{DD2}$ to output is then determined by

(8) $$T=\left(R_{o}\|R\right)C_{L}$$

**Figure 5:**
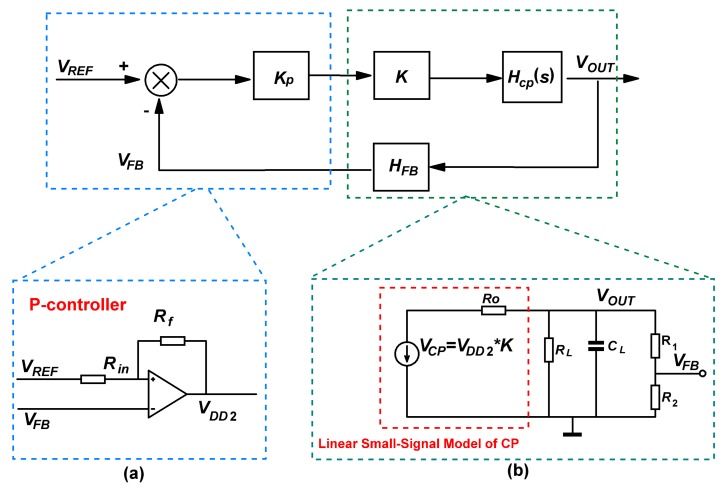
Block diagram of the closed-loop model. (**a**) P-controller, (**b**) linearized charge pump small-signal mode.

The block diagram of the closed loop is shown in Figure 5. $K_{p}$ is the gain of the P-controller. Unlike using VCO circuits, the realization of the regulation is by using a full differential amplifier to change the amplitude of the clocks according to the difference between $V_{REF}$ and $V_{FB}$, which is easier to design and has better linearity performance.

The loop transfer function, i.e., the transfer function from the reference to the output can be expressed as

(9) $$\frac{V_{OUT}\left(s\right)}{V_{REF}\left(s\right)}=\frac{K_{p}H_{CP}\left(s\right)}{1+K_{p}H_{FB}H_{CP}\left(s\right)}=\frac{K_{p}K_{CP}}{1+K_{p}K_{CP}H_{FB}}\cdot \frac{1}{\frac{T}{1+K_{p}K_{CP}H_{FB}}s+1}$$

After closed-loop regulation, the time constant of the system turns to be

(10) $$T^{\prime }=\frac{T}{1+K_{p}K_{CP}H_{FB}}$$

which reduces the start-up time of the system. Besides, the gain from reference to the output are determined by

(11) $$K^{\prime }=\frac{K_{p}K_{CP}}{1+K_{p}K_{CP}H_{FB}}$$

Assuming $K_{p}K_{CP}H_{FB}\gg 1$, the gain $K^{\prime }$ is given by

(12) $$K^{\prime }\approx \frac{1}{H_{FB}}$$

Thus, the output voltage of the closed-loop charge pump is

(13) $$V_{OUT}\approx \frac{1}{H_{FB}}\cdot V_{REF}=\left(1+\frac{R_{1}}{R_{2}}\right)\cdot V_{REF}$$

Considering that the temperature coefficients of $R_{1}$ and $R_{2}$ are almost equal in the same die, the output voltage of the closed-loop charge pump is insensitive to temperature and process variation.

## 3. Experimental Results

**Figure 6:**
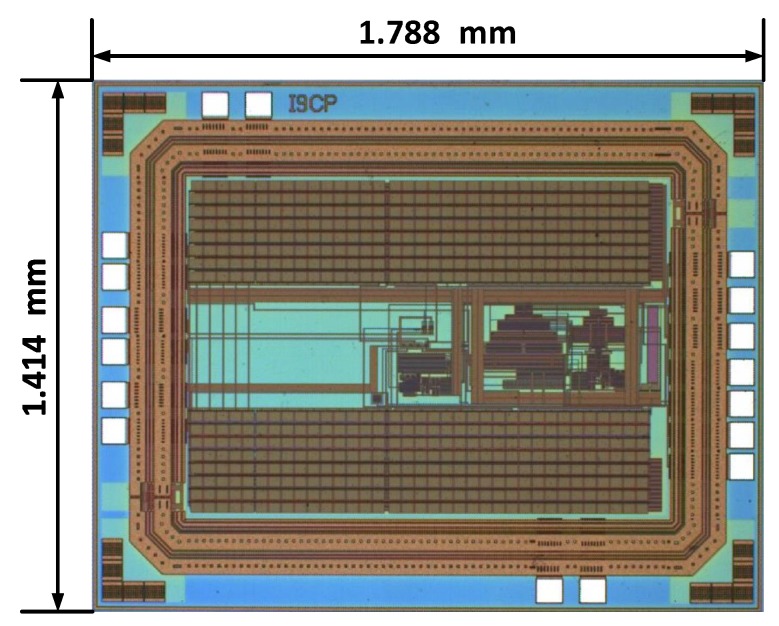
Micro-photograph of the charge pump.

The proposed charge pump was fabricated in SMIC 0.18-μm standard CMOS process and the chip micro-photograph is shown in Figure 6. The area of the whole chip including I/O pads is 2.53 mm${}^{2}$, most of which is occupied by on-chip pumping metal-insulator-metal (MIM) capacitors (72 pF each).

**Figure 7:**
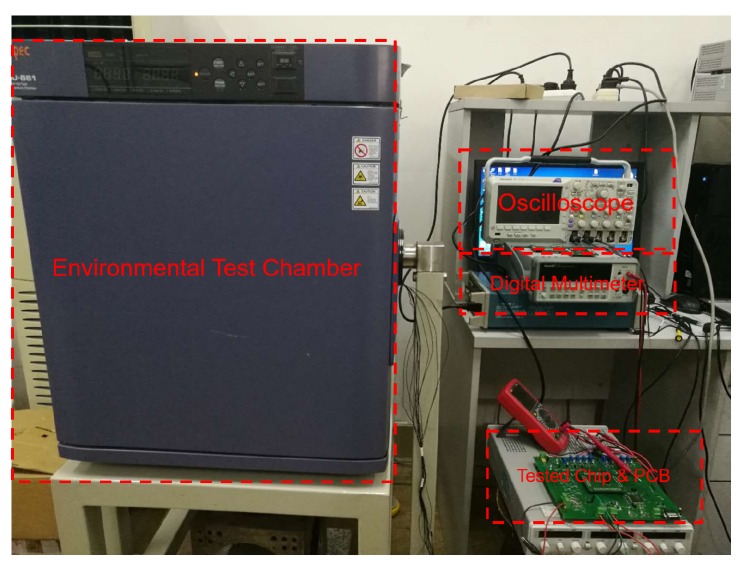
Experimental setup for testing the charge pump.

Figure 7 shows the experimental setup for testing the charge pump’s performance. We used the $7\frac{1}{2}$-digit Keithley 2010 Multimeter to measure the output voltage. The DC power supply was 5 V. The charge pump was driven by differential clocks with a frequency of 10 kHz.

**Figure 8:**
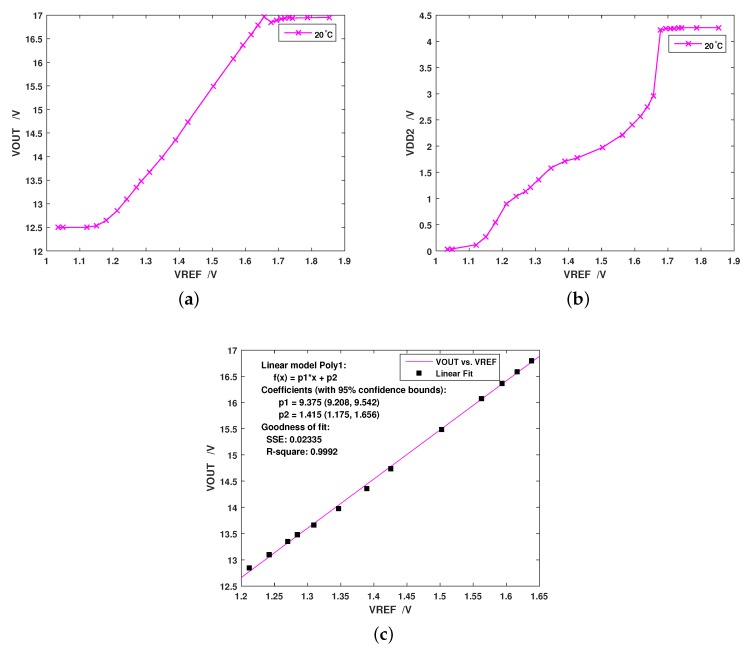
Measured output of the charge pump at 20 ${}^{\circ }C$: (**a**) the tested graph of output vs. reference voltage; (**b**) the tested controller voltage $V_{DD2}$ vs. reference; and (**c**) regression of the linear interval in (**a**).

The charge pump’s output voltage varying with reference voltage was first tested, and the result is depicted in Figure 8a. We found that the output could not keep increasing along with the reference, since the maximum value of $V_{DD2}$ was restricted by the output swing of the amplifier in the P-controller, as Figure 8b shows. The maximum voltage that the charge pump could reach is 16.95 V at 20 ${}^{\circ }C$. As the reference voltage varied from 1.2 V to 1.65 V, the output voltage showed good linearity against the reference, as shown in Figure 8c. This indicates that the output voltage can be adjusted proportionally from around 13 V to 16.95 V.

**Figure 9:**
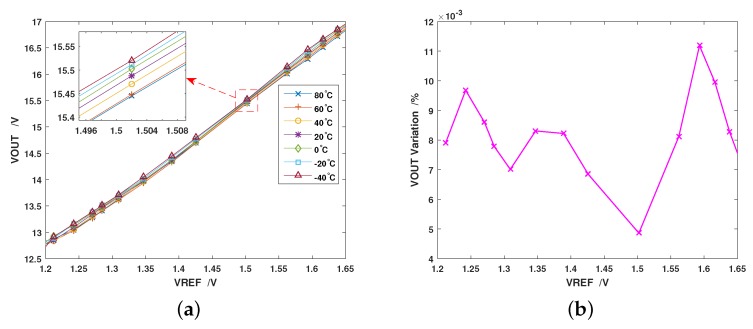
Measured output of the charge pump under different temperature conditions: (**a**) the tested graph of output vs. reference voltage in the linear interval; and (**b**) the maximum variation of the output voltage from $-40{\;}^{\circ }C$ to $80{\;}^{\circ }C$.

Then, the charge pump’s temperature characteristics were tested with the PCB (printed circuit board) placed in an Espec SU-661 Environmental Test Chamber. The $V_{OUT}$ vs. $V_{REF}$ graph was sampled every 20 ${}^{\circ }C$ from −40∼80 degrees, and the results are shown in Figure 9a (only the linear interval displayed). We can see that the charge pump maintained its linearity under every temperature condition, and the output voltage had little fluctuation at each reference level. Figure 9b shows the output variation vs. reference voltage, which indicates that the output of this charge pump varied less than 1.1% in the whole temperature range.

Table 1 compares the charge pump in this work with previous studies. Hong and El-Gamal [29] realized on-chip HV up to the breakdown limit of single n-well/substrate in a standard technology via PMOS-only transistors, and without closed-loop operation, the charge pump performed a 10% variance in the output from $-55∼100{\;}^{\circ }$C. Shen et al. [19] adopted the idea of triple-well devices and implemented a digital logic to control the charging clock to regulate the output voltage, but non-linearity and discontinuity of digital approach limited its application. Aaltonen and Halonen [22] designed a charge pump for gyroscope in HV CMOS process with closed-loop operation by voltage-controlled oscillator (VCO) controlling the pumping frequency, but it only boosted the output to about 10 V. The HV output in this work achieved 16.95 V with variation less than 1.1% in $-40∼80{\;}^{\circ }C$ range via controlling the pumping clock amplitude. It was fabricated in a standard CMOS process using triple-well devices to boost beyond p-n junction reverse breakdown limitation, making it compatible with gyroscope ASICs (application specific integrated circuits).

## 4. Conclusions

A high-voltage charge pump fabricated in a 0.18-μm standard CMOS process is presented in this paper. The output voltage has a linear adjustment range versus reference voltage from around 13 V to 16.95 V. Moreover, by applying closed-loop control, the charge pump’s output is regulated from −40 to 80 degrees, with the voltage deviation less than 1.1%. Therefore, the proposed charge pump in this paper can generate sufficient and stable voltage for actuation and control of micro-gyroscopes to improve the sensors’ accuracy. Besides, the proposed charge pump does not require special HV device, which implies its potential to be integrated into gyroscope interface with other analog and digital modules to build a single-chip gyroscope measurement system. Future works may include introducing other closed-loop control methods to reach lower temperature variations, combining it with a bandgap reference as $V_{REF}$, and utilizing a practical gyroscope interface with this temperature-insensitive charge pump.

## Figures and Tables

**Table 1:** Performance comparison with previous researches.

Parameter	This Work	[19]	[22]	[29]
Process	0.18-μm CMOS	0.13-μm CMOS	0.35-μm HV CMOS	0.18-μm CMOS
Supply Voltage	5 V	1.8 V	3.6 V	1.2 V
Maximum Output	16.95 V	22 V	10.4 V	14.8 V
Stage Capacitor	72 pF	N/A	1.6–1.1 pF	5.4 pF
Closed-loop Approach	Clock Amplitude	Digital Control	Clock Frequency	Open-loop
Temperature Variation	1.1% ($-40∼80{\;}^{\circ }$C)	N/A	N/A	10% ($-55∼100{\;}^{\circ }$C)
Area	2.52 mm${}^{2}$	0.149 mm${}^{2}$	0.14 mm${}^{2}$	N/A

## References

[1] Finkbeiner S. MEMS for automotive and consumer electronics. Proceedings of the 2013 European Solid-State Device Research Conference (ESSDERC).

[2] Zhang T., Zhou B., Yin P., Chen Z., Zhang R. (2016). Optimal Design of a Center Support Quadruple Mass Gyroscope (CSQMG). Sensors.

[3] Sharma A., Zaman M.F., Ayazi F. (2009). A Sub-0.2°/hr Bias Drift Micromechanical Silicon Gyroscope with Automatic CMOS Mode-Matching. IEEE J. Solid-State Circuits.

[4] Sung W.K., Dalal M., Ayazi F. A mode-matched 0.9 MHz single proof-mass dual-axis gyroscope. Proceedings of the 2011 16th International Solid-State Sensors, Actuators and Microsystems Conference.

[5] Woo J., Boyd C., Cho J., Najafi K. Ultra-low-noise transimpedance amplifier for high-performance MEMS resonant gyroscopes. Proceedings of the 2017 19th International Conference on Solid-State Sensors, Actuators and Microsystems (TRANSDUCERS).

[6] Saukoski M., Aaltonen L., Salo T., Halonen K.A.I. (2008). Interface and control electronics for a bulk micromachined capacitive gyroscope. Sens. Actuators A Phys..

[7] Chen W., Fu Q., Yuan Y., Song R., Li Y., Liu X. Design of charge-pump phase locked loop in micro-inertial sensor. Proceedings of the 2011 Academic International Symposium on Optoelectronics and Microelectronics Technology.

[8] Chu Y., Dong J., Chi B., Liu Y. (2016). A Novel Digital Closed Loop MEMS Accelerometer Utilizing a Charge Pump. Sensors.

[9] Guo Z.Y., Yang Z.C., Zhao Q.C., Lin L.T., Ding H.T., Liu X.S., Cui J., Xie H., Yan G.Z. (2009). A lateral-axis micromachined tuning fork gyroscope with torsional *Z*-sensing and electrostatic force-balanced driving. J. Micromech. Microeng..

[10] Marx M., Rombach S., Nessler S., de Dorigo D., Manoli Y. (2019). A 141-μW High-Voltage MEMS Gyroscope Drive Interface Circuit Based on Flying Capacitors. IEEE J. Solid-State Circuits.

[11] Rombach S., Marx M., Nessler S., de Dorigo D., Maurer M., Manoli Y. (2016). An Interface ASIC for MEMS Vibratory Gyroscopes with a Power of 1.6 mW, 92 dB DR and 0.007 °/s/$\sqrt{\mathrm{Hz}}$ Noise Floor Over a 40 Hz Band. IEEE J. Solid-State Circuits.

[12] Tanzawa T. (2013). System overview and key design considerations. On-Chip High-Voltage Generator Design.

[13] Prandi L., Caminada C., Coronato L., Cazzaniga G., Biganzoli F., Antonello R., Oboe R. A low-power 3-axis digital-output MEMS gyroscope with single drive and multiplexed angular rate readout. Proceedings of the 2011 IEEE International Solid-State Circuits Conference.

[14] Emira A., AbdelGhany M., Elsayed M., Elshurafa A.M., Sedky S., Salama K.N. (2013). All-pMOS 50-V Charge Pumps Using Low-Voltage Capacitors. IEEE Trans. Ind. Electron..

[15] Trusov A.A., Prikhodko I.P., Rozelle D.M., Meyer A.D., Shkel A.M. 1 PPM precision self-calibration of scale factor in MEMS Coriolis vibratory gyroscopes. Proceedings of the 2013 Transducers Eurosensors XXVII: The 17th International Conference on Solid-State Sensors, Actuators and Microsystems (TRANSDUCERS EUROSENSORS XXVII).

[16] Dalal M. (2012). Low Noise, Low Power Interface Circuits and Systems for High Frequency Resonant Micro-Gyroscopes. Ph.D. Thesis.

[17] Aaltonen L., Kalanti A., Pulkkinen M., Paavola M., Kämäräinen M., Halonen K. A 4.3 mm^2^ ASIC for a 300 °/s 2-axis capacitive micro-gyroscope. Proceedings of the 2010 ESSCIRC.

[18] Lee J.Y., Kim S.E., Song S.J., Kim J.K., Kim S., Yoo H.J. (2006). A Regulated Charge Pump with Small Ripple Voltage and Fast Start-Up. IEEE J. Solid-State Circuits.

[19] Shen B., Bose S., Johnston M.L. On-chip high-voltage SPAD bias generation using a dual-mode, closed-loop charge pump. Proceedings of the 2017 IEEE International Symposium on Circuits and Systems (ISCAS).

[20] Hoque M.R., McNutt T., Zhang J., Mantooth A., Mojarradi M. A high voltage Dickson charge pump in SOI CMOS. Proceedings of the IEEE 2003 Custom Integrated Circuits Conference.

[21] Ker M., Chen S. (2007). Ultra-High-Voltage Charge Pump Circuit in Low-Voltage Bulk CMOS Processes with Polysilicon Diodes. IEEE Trans. Circ. Syst. II Express Briefs.

[22] Aaltonen L., Halonen K. On-chip charge-pump with continuous frequency regulation for precision high-voltage generation. Proceedings of the 2009 Ph.D. Research in Microelectronics and Electronics.

[23] Zhang M., Llaser N. On-Chip High Voltage Generation with standard process for MEMS. Proceedings of the 2007 14th IEEE International Conference on Electronics, Circuits and Systems.

[24] Ismail Y., Lee H., Pamarti S., Yang C.K. A 34V charge pump in 65nm bulk CMOS technology. Proceedings of the 2014 IEEE International Solid-State Circuits Conference Digest of Technical Papers (ISSCC).

[25] Shirane A., Ito H., Ishihara N., Masu K. A 21 V output charge pump circuit with appropriate well-bias supply technique in 0.18 μm Si CMOS. Proceedings of the 2011 International SoC Design Conference.

[26] Dickson J.F. (1976). On-chip high-voltage generation in MNOS integrated circuits using an improved voltage multiplier technique. IEEE J. Solid-State Circuits.

[27] Ismail Y., Lee H., Pamarti S., Yang C.K.K. (2017). A 36-V 49% Efficient Hybrid Charge Pump in Nanometer-Scale Bulk CMOS Technology. IEEE J. Solid-State Circuits.

[28] Tanzawa T., Tanaka T. (1997). A dynamic analysis of the Dickson charge pump circuit. IEEE J. Solid-State Circuits.

[29] Hong D., El-Gamal M. Low operating voltage and short settling time CMOS charge pump for MEMS applications. Proceedings of the 2003 International Symposium on Circuits and Systems, ISCAS 2003.

